# Characteristics Peripheral Blood IgG and IgM Heavy Chain Complementarity Determining Region 3 Repertoire before and after Immunization with Recombinant HBV Vaccine

**DOI:** 10.1371/journal.pone.0170479

**Published:** 2017-01-23

**Authors:** Long Ma, Xiaomei Wang, Xiaoying Bi, Jiezuan Yang, Bin Shi, Xiaoyan He, Rui Ma, Qingqing Ma, Xinsheng Yao

**Affiliations:** 1 Department of Immunology, Research Center for Medicine & Biology, Innovation & Practice Base for Graduate Students Education, Zunyi Medical University, Zunyi, Guizhou, China; 2 The First Hospital of Hunan University of Chinese Medicine, Changsha, Hunan, China; 3 State Key Laboratory for Diagnosis and Treatment of Infectious Diseases, Collaborative Innovation Center for Diagnosis and Treatment of Infectious Diseases, The First Affiliated Hospital, College of Medicine, Zhejiang University, Hangzhou, Zhejiang, China; 4 Department of Laboratory Medicine, Zunyi Medical University, Zunyi, Guizhou, China; Centre de Recherche en Cancerologie de Lyon, FRANCE

## Abstract

Immunization with recombinant HBV vaccine induces specific immune responses in human causing B lymphocytes to produce protective HBsAb, and to form memory B lymphocytes, thereby facilitating HBV immunity in the body. However, B lymphocytes heterogeneity and characteristics are not fully elucidated. In this study, we conducted high-throughput sequencing of BCR heavy chain CDR3 repertoires in 3 healthy volunteers before and after the third immunization with recombinant HBV vaccine. We used Roche 454 Genome Sequencer FLX system to perform a comparative analysis of IgM and IgG H chain CDR3 repertoires. First, we found that the diversity of IgG H chain CDR3 repertoires was 1/6 of IgM on average. Moreover, after the third immunization with HBV vaccine, the diversity of IgG H chain CDR3 repertoires was 1/26 of IgM on average. Second, we detected relatively high levels of HBsAbs in all the healthy volunteers after immunization with HBV vaccine. The volunteers shared a small number of CDR3 sequences before and after immunization, and among each other. However, we did not find completely identical BCR H chain CDR3 amino acid sequences in these volunteers. Lastly, after immunization with recombinant HBV vaccine, the volunteers showed high frequency of IgG H chain CDR3 amino acid sequences mostly resulting from rearrangements of IGHV, IGHD and IGHJ, suggesting that the mechanism of high frequency CDR3 generation might be associated with the maturation of IgG affinity (somatic hypermutation) during the recombinant HBV vaccine-induced B lymphocyte responses. This study identified the characteristics and changes of BCR CDR3 repertoires before and after immunization with HBV vaccine, and evaluated the performance of the sequencing technology for this application. Our findings provide a basis for further research in B lymphocyte generated HBsAb heterogeneity and monitoring the maintenance of memory B lymphocytes.

## Introduction

Immunization with recombinant hepatitis B virus (HBV) vaccine helps prevent the incidence of HBV infection and hepatitis B. Since 1990s, China has introduced the immunization program for HBV vaccination, which has rapidly reduced the carriage rate of hepatitis B surface antigen (HBsAg). This program has greatly prevented HBV infection and also reduced the number of new cases [1,2]. In China, the recombinant HBV vaccine commonly containing 5ug HBsAg. Following immunization in humans, the fragments captured and hydrolyzed by antigen presenting cells combine with major histocompatibility complex class II (MHC-II) molecules to form MHC-II antigen complexes, which are then secreted to the cell surface and interact with CD4^+^ T helper cell surface antigen to stimulate CD4^+^ T cells activation and transformation into Th2 cells. Th2 cells react with B lymphocytes that can recognize HBsAg, and the activated B lymphocytes then differentiate into plasma cells and secrete protective HBsAb. Additionally, B lymphocytes can not only act as antigen-presenting cells to directly recognize HBsAg, but also interact with activated Th2 cells to produce HBsAb [3].

However, the heterogeneity of B lymphocytes which secrete hepatitis B surface antibody (HBsAb) in the human body as well as the characteristics and longevity of memory B lymphocytes have not been fully elucidated. Maeda et al. studied the B cell receptor (BCR) sequence associated with the response to HBV vaccination and the synthesis of anti-HBsAg monoclonal antibodies, and developed HBsAb transgenic tomatoes [4–6]. Several other studies have utilized BCR sequencing and phage display technology to detect HBsAbs with high affinity to HBsAg [7–9]. Yao et al. and Yang et al. used gene melting spectral pattern (GMSP) assay to detect clonal expansion in CD4^+^T and CD8^+^T cells in patients with acute HBV infection. These studies suggested that T cells act as bystander activators with constant expression of the complementarity determining region 3 (CDR3) motif in response to HBV virus, and assist antigen-specific B lymphocyte responses [10,11]. These studies selected and screened a limited number of anti-HBV antibody sequences. By focusing on the BCR gene, these studies provided new research strategies to study the mechanism of HBsAb secretion by B lymphocytes, and the maintenance of memory B lymphocytes.

In the recent 5 years, high-throughput sequencing (HTS) has been able to preliminarily assess the basic BCR repertoire characteristics of an individual. And has been used to analyzed the antibody repertoires after vaccination. Galson et al. analyzed IgG and IgM heavy chain repertoires from circulating B cells before and after HBV vaccination using Illumina sequencing, their results showed the HTS could help us to understand immune responses to disease and vaccination, they also reported that the repertoires appear to correlate with the PCs appearing at 7 days after immunization, and has a more similar kinetic to appearance of memory cells at 21 days after immunization [12]. They also reported that focusing on the vaccine-specific repertoires and subsequent doses were essential to find high affinity B cell clones to specific antigen [13]. But there is no research to analyzed the compositions and characteristics of IgM and IgG chain CDR3 repertoires. In this study, we selected 3 healthy volunteers for immunization with recombinant HBV vaccine, and used Roche 454 Genome Sequencer FLX system high-throughput sequencing (454 GS-FLX HTS) to analyze the BCR heavy (H) chain CDR3 repertoires before and after immunization. We comparatively analyzed the compositions and characteristics of IgM and IgG chain CDR3 repertoires to provide basic data and new research methodologies for investigating the heterogeneity of B lymphocytes on generating HBsAb as well as monitoring the maintenance of memory B lymphocytes.

## Materials and Methods

### Immunization of recombinant HBV vaccine and serological tests before and after immunization in the healthy volunteers

The healthy volunteers recruited for this study were medical students without autoimmune diseases and malignant tumors, and had signed the written informed consents before participating in the study. This program was conducted in 2008 at Zunyi Medical College, Guizhou, China. All the research protocols were approved by the ethics committee of Zunyi Medical College. All participants underwent Roche electrochemiluminescence (ECL) quantitative detection to screen the 5 HBV hepatitis markers (serological tests were completed by laboratory of the Affiliated Hospital of Zunyi Medical College). Inclusion criteria: participants with negative results in the screening of HBV hepatitis markers were included in the immunization with recombinant HBV vaccine. Three healthy volunteers, including Y (male, 23 years old, Dong minority), L (male, 24 years old, Han Chinese), and Z (male, 23 years old, Dong minority), were selected for the immunization program. They were immunized with recombinant HBV vaccine according to the standard immunization program (intramuscular injection 3 times at months 0, 1, and 6), and the levels of HBsAb were detected using Roche ECL assay. Peripheral blood (15 ml) was separately collected one day before entire immunization (one day before the first immunization) and 14 days after the third immunization with recombinant HBV vaccine. The 6 samples from Y’s pre-immunization, Y’s post-immunization, L’s pre-immunization, L’s post-immunization, Z’s pre-immunization, and Z’s post-immunization were labelled as MID1 = YQ, MID2 = YH, MID3 = LQ, MID4 = LH, MID5 = ZQ, and MID6 = ZH, respectively.

### Human leukocyte antigen (HLA) typing

Whole blood (5 ml) collected from the volunteers in the immunization program was placed in collection tubes containing EDTAk2 anticoagulant to perform HLA-A and HLA-DR genotyping using PCR-sequence based typing (SBT) method (BGI Shenzhen, Shenzhen, China). Nine groups of group-specific primers with additional modification at the 5’ end were used to amplify the target DNA, and to sequence the amplified PCR products. The sequenced samples underwent HLA typing according to the HLA sequence database.

### Peripheral blood mononuclear cell (PBMC) isolation, total RNA extraction and cDNA synthesis

PBMC was isolated from heparin treated peripheral blood using density gradient centrifugation. Total RNA was extracted from the PBMCs according to the manufacturer’s protocol for the total RNA extraction kit (Omega Bio-Tek). The total RNA was then reverse transcribed into cDNA using Oligo dT18 according to the manufacturer’s protocol for reverse transcription kit (MBI, Fermentas).

### PCR amplification of human BCRH chain CDR3 repertoires

Six BCR HV upstream primers and 2 BCR HC downstream primers (Table 1) were designed by Thermo Fisher Scientific (Shanghai, China). The 5’ end of each primer had a unique MID sequence consisting of 10 nucleotides. The primers were used to amplify BCR H chain CDR3 repertoires of different samples. The reaction system required 20 μl total volume of reaction mixture, containing 1μl cDNA template, 1μl(50μmol/L) IGHV corresponding upstream primer, 1μl(50μmol/L) IgM/IgG downstream primer, 10 μl FQ-PCR master mix, and 7 μl distilled water. PCR reaction conditions included an initial denaturing cycle at 96°C for 30 s, followed by 35 cycles of denaturation, annealing, and extension at 96°C for 10 s, 58°C for 15 s, and 72°C for 30 s, respectively, and subsequently an extension cycle at 72°C for 5 min. PCR products were recovered using agarose gel purification kit (Qiagen) in accordance with the manufacturer’s protocol. Concentration and total yield of each sample were measured using capillary electrophoresis before the HTS in Tongji-SCBIT Biotechnology Corporation, Ltd. (Shanghai, China).

**Table 1:** Human IGHV/CHM/CHG primer sequences and barcode sequences.

Name of primer	Sequences(5’~3’)	Barcode	Sequences(5’~3’)
**IGHV1**	AGGGCTTGAATGGACAGG	**MID1**	CGTGTCTCTA
**IGHV2**	CTCCAAGGACACCTCCAAG	**MID2**	CTCGCGTGTC
**IGHV3**	CCAGGGACAACGCCAAGAAC	**MID3**	TAGTATCAGC
**IGHV4**	GCTGAGCTCTGTGACCGC	**MID4**	TCTCTATGCG
**IGHV5**	ATCTCAGCCGACAAGTCCATCAG	**MID5**	TGATACGTCT
**IGHV6**	TGTGACTCCCGAGGACACGGC	**MID6**	TACTGAGCTA
**CHM**	CAGGAGACGAGGGGGAAAAGG		
**CHG**	CACCGTCACCGGTTCGGGG		
**GAPDH Anti-sense**	GGTGAAGGTCGGTGTGAACG		
**GAPDH sense**	CTCGCTCCTGGAAGATGGTG		

### 454 GS-FLX HTS of BCR H chain CDR3 repertoires and analysis

Roche 454 GS-FLX HTS was conducted by Tongji-SCBIT Biotechnology Corporation. Sequence screening process of CDR3 repertoires: (1) exclude sequences without single and unique MID sequence tags. (2) after the initial screening process, the CDR3 sequence was distinguished using HV/HC primers and IgM (VTCSSGSASAP)/IgG (VTVSSASTKGP) amino acid sequences of the constant region domain. The resulting sequences of IgM and IgG H chain CDR3 repertoires after sorting were further screened by IMGT/High V Quest: (1) exclude sequences with no functionality finding and unknown functionality, (2) exclude sequence with < 85% V region-identity, (3) exclude sequence with junction frame equal to null, and (4) exclude sequence with non-C amino acid at 104-site or non-F amino acid at 118-site in AA junction. After the screening process, sequence containing stop code or pseudogenes, or its junction frame as out-of-frame in the functionality comment was defined as unproductive sequence; while the remaining sequence was defined as productive sequence. Productive sequences were grouped into clonotypes (grouping of sequencing reads with identical Variable segment, Joining segment and CDR3 amino acid sequence).

Productive sequences were analyzed according to the composition and characteristics of inverse Simpson’s index (1/DS), overlapping CDR3, and high frequency CDR3 [14–17]. The diversity of clonotypes was calculated using inverse Simpson's index, using the formula *D*_s_ = 1 − Σ[*n*_i_(*n*_i_ − 1)]/[*N*(*N* − 1)], where *n*_i_ is the clone size of the *i*th clonotype and *N* is the total number of sequence reads [16,17].

## Results

### HLA typing and HBsAb detection

Table 2 shows the results of HLA-A and HLA-DR typing as well as HBsAg, hepatitis B e antigen (HBeAg), hepatitis B e antibody (HBeAb), hepatitis B core antibody (HBcAb), and HBsAb in the selected 3 healthy volunteers (Y, L, and Z) before and after immunization with recombinant HBV vaccine. The volunteers had a part of the HLA-A site (A*0207) that was identical, whereas the HLA-DR3 site was different. The HBsAg and HBsAb screening of the 3 healthy volunteers before immunization with recombinant HBV was negative, while HBsAb levels reached the normal response levels (> 100 IU/L) 6 months after immunization.

**Table 2:** Demographical and clinical characteristics of 3 volunteers.

Samples	Age(years)	Gender	HLA-A type	HLA-DR type	HBsAg(COI)	HBsAb(mIU/ml)	HBeAg(COI)	HBeAb(COI)	HBcAb(COI)	HBsAb(mIU/ml)
**Y**	23	female	A*0203 A*0207	DRB1*0803DRB1*0803	0.585	<<2.000	0.091	1.560	1.970	147.000
**L**	24	female	A*2402 A*0201	DRB1*0403DRB1*1202	0.553	<<2.000	0.094	1.710	2.010	251.600
**Z**	23	female	A*0301 A*0207	DRB1*0901DRB1*0701	0.473	7.370	0.108	1.630	2.120	107.600

### BCR H chain CDR3 repertoires preparation and HTS

The BCR H chain CDR3 sequences from the 6 samples of the 3 healthy volunteers before and after the immunization with recombinant HBV vaccine were amplified by PCR. The purified PCR products were identified using agarose gel electrophoresis. Capillary electrophoresis showed that the concentration of each sample was above 50 ng/μl, and the total amount of DNA was above 6 μg (the total DNA amounts of the 6 samples in the 454 GS-FLX HTS were identical).

**Fig 1:**
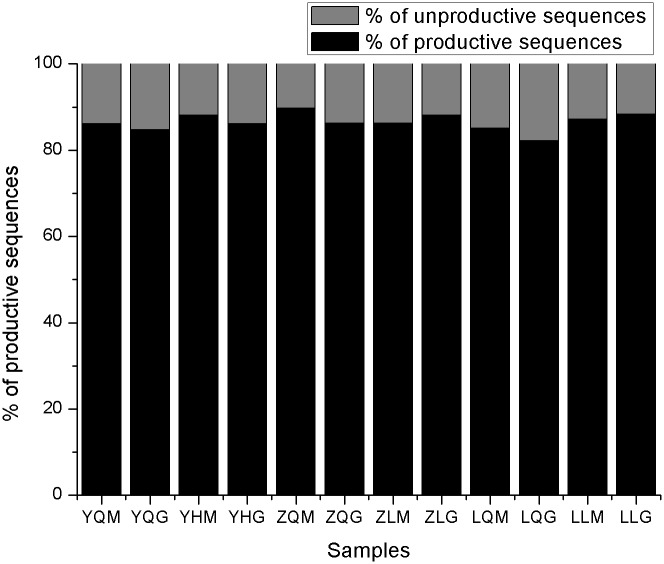
The percentage of productive sequences in the repertoires of IgM and IgG before and after immunization with HBV vaccines in 3 volunteers.

BCR H chain CDR3 repertoires total, productive, unproductive, IgG and IgM H chain CDR3 sequences were all identical in the 3 healthy volunteers before and after immunization with recombinant HBV vaccine (Table 3, Fig 1).

**Table 3:** Characteristics of sequence data.

Samples	Total	AnalysisSequences	Productive sequences	Unproductive sequences	Productive sequences(%)
YQM	1290	606	522^a^/427^b^	84	86.14
YQG	1645	1120	949^a^/480^b^	171	84.73
YHM	2450	2047	1804^a^/1446^b^	243	88.13
YHG	1566	1128	972^a^/699^b^	156	86.17
ZQM	1444	890	798^a^/494^b^	92	89.66
ZQG	1431	372	321^a^/221^b^	51	86.29
ZHM	1051	625	539^a^/478^b^	86	86.24
ZHG	1056	707	623^a^/398^b^	84	88.12
LQM	2082	1357	1154^a^/825^b^	203	85.04
LQG	1036	605	497^a^/238^b^	108	82.15
LHM	1750	883	770^a^/649^b^	113	87.20
LHG	3090	1706	1508^a^/429^b^	198	88.39

^a^ Total productive sequences.

^b^ Unique productive sequences.

### Diversity of IgM and IgG H chain CDR3 repertoires before and after immunization with recombinant HBV vaccine

**Fig 2:**
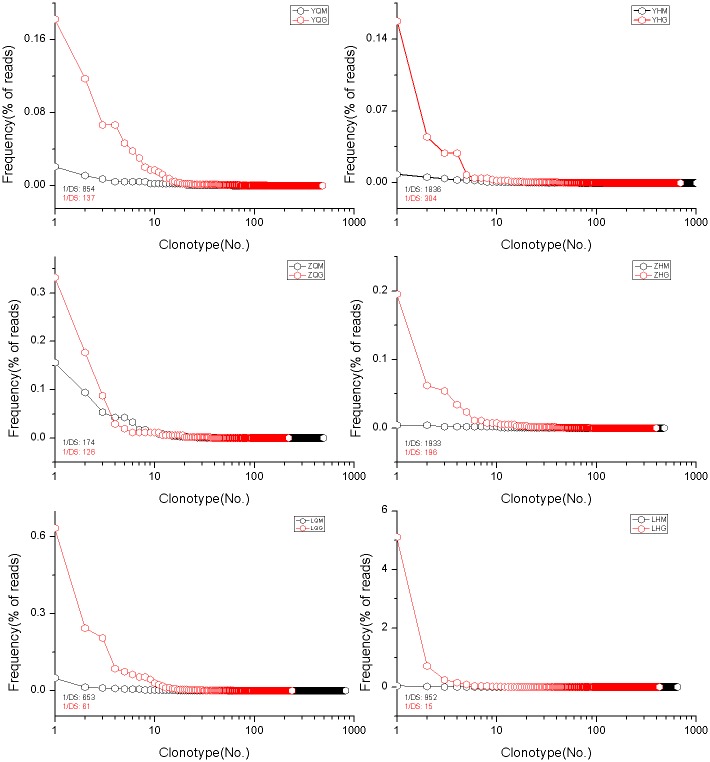
The diversity of repertoires of IgM and IgG before and after immunization with HBV vaccines in 3 volunteers.

Fig 2 shows the diversity analysis of IgM and IgG H chain CDR3 repertoires of the 3 healthy volunteers before and after immunization with recombinant HBV vaccine. Comparing IgM and IgG in the same sample, we found that the diversity of IgG H chain CDR3 repertoires was 1/6 of the diversity of IgM H chain CDR3 repertoires on average before immunization with recombinant HBV vaccine. The diversity of IgG H chain CDR3 repertoires was 1/26 of the diversity of IgM H chain CDR3 repertoires on average after immunization with recombinant HBV vaccine.

### IgM and IgG H chain CDR3 overlapping sequences before and after immunization with recombinant HBV vaccine

Table 4 demonstrates the results of overlapping IgM and IgG H chain CDR3 repertoires in the 3 healthy volunteers before and after immunization with recombinant HBV vaccine. The proportions of the common sequence in different samples were rather low. The CDR3 sequence was not identical in the 3 healthy volunteers. Furthermore, we obtained a high overlapping proportion of IgM and IgG H chain CDR3 repertoires in the same individual and at the same time (all above 1% and up to 15.84%). A small number of public CDR3 sequences were found in the 3 healthy volunteers before and after immunizations, so as among each other. However, no identical IgG/IgM H chain CDR3 sequence was found among the 3 healthy volunteers.

**Table 4:** The amount and frequency of sequences that overlap in the repertoires of IgM and IgG before and after immunization with HBV vaccines in 3 volunteers.

	YQM	YQG	YHM	YHG	ZQM	ZQG	ZHM	ZHG	LQM	LQG	LHM	LHG
YQM	426	17(3.99%)	15(1.04%)	1(0.14%)	1(0.20%)	0(0)	2(0.42%)	1(0.25%)	2(0.24%)	1(0.42%)	2(0.31%)	1(0.23%)
YQG	17(3.54%)	480	3(0.21%)	7(1.00%)	0(0)	5(2.26%)	1(0.21%)	2(0.50%)	0(0)	3(0.13%)	3(0.46%)	7(1.63%)
YHM	15(3.52%)	3(0.63%)	1446	37(5.29%)	1(0.20%)	1(0.45%)	1(0.21%)	1(0.25%)	3(0.36%)	2(0.84%)	5(0.77%)	1(0.23%)
YHG	1(0.23%)	7(1.46%)	37(2.56%)	699	0(0)	1(0.45%)	0(0)	2(0.50%)	0(0)	2(0.84%)	2(0.31%)	1(0.23%)
ZQM	1(0.23%)	0(0)	1(0.07%)	0(0)	494	35(15.84%)	3(0.63%)	1(0.25%)	4(0.48%)	1(0.42%)	5(0.77%)	3(0.70%)
ZQG	0(0)	5(1.04%)	1(0.07%)	1(0.14%)	35(7.09%)	221	1(0.21%)	1(0.25%)	0(0)	0(0)	1(0.15%)	4(0.93%)
ZHM	2(0.47%)	1(0.21%)	1(0.07%)	0(0)	3(0.61%)	1(0.45%)	478	12(3.02%)	0(0)	1(0.42%)	2(0.31%)	0(0)
ZHG	1(0.23%)	2(0.42%)	1(0.07%)	2(0.29%)	1(0.20%)	1(0.45%)	12(2.51%)	398	0(0)	1(0.42%)	0(0)	0(0)
LQM	2(0.47%)	0(0)	3(0.21%)	0(0)	4(0.81%)	0(0)	0(0)	0(0)	825	45(18.91%)	3(0.46%)	0(0)
LQG	1(0.23%)	3(0.63%)	2(0.14%)	2(0.29%)	1(0.20%)	0(0)	1(0.21%)	1(0.25%)	45(5.45%)	238	1(0.15%)	3(0.70%)
LHM	2(0.47%)	3(0.63%)	5(0.35%)	2(0.29%)	5(1.01%)	1(0.45%)	2(0.42%)	0(0)	3(0.36%)	1(0.42%)	649	21(4.90%)
LHG	1(0.23%)	7(1.46%)	1(0.07%)	1(0.14%)	3(0.61%)	4(1.81%)	0(0)	0(0)	0(0)	3(0.13%)	21(3.24%)	429

### High frequency CDR3 amino acid sequence analysis of IgG H chain CDR3 repertoires after immunization with recombinant HBV vaccine

As shown in Table 5, in the IgG H chain CDR3 repertoires, the proportion of sequence of CDR3 frequency > 0.5% was relatively high and consistent among the 3 healthy volunteers after immunization with recombinant HBV vaccine. In the same sample, high frequency sequence families had access to wide distribution, and their isoelectric point (pI) and average hydrophilic grand average of hydropathicity (GRAVY) distributions were relatively wide. In the high frequency expressed CDR3 sequence, the IgG receptor library of the 3 healthy volunteers after immunization with recombinant HBV vaccine showed different IGHV and IGHJ family rearrangements to generate the same amino acid sequence of CDR3 (Table 6). In this study, we first selected part of the sequence and used an integrated software IMGT/V-quest to further analyze the accession and point mutations of the sequence family. Our finding showed that the CDR3 region had high frequency of mutations. Through specific point mutation, we found that different families’ accession sequence contained identical CDR3 amino acid sequence.

**Table 5:** Characteristics of high frequency CDR3 sequences (>0.5%) in the IgG repertoires after immunization with HBV vaccines in 3 volunteers.

_______	_________	High cloned sequences				
Sample	Frequency	___	Peptide	V(D)J	GRAVY	pI
YHG	4.01%	1	CARGGSGSKAGLFDSW	V1-69 D6-19 J4;V6-1 D6-19 J4	-0.256	8.22
	2.16%	2	CARSGSGWKAGLFDSW	V6-1 D6-19 J4	-0.287	8.22
	1.75%	3	CARRDYGGNPLRPWGYYYGMDVW	V5-51 D4-23 J6	-0.939	8.15
	1.75%	4	CARWGPVELLSDYW	V1-18 D2-21 J4; V1-3 D2-21 J4	-0.093	4.37
	0.93%	5	CGRHRGGEVATMGAFDIW	V4-39 D5-24 J2	-0.161	6.74
	0.72%	6	CARDLNSSYLYNWLDPW	V1-46 D6-13 J5;V1-18 D6-13 J5	-0.612	4.21
	0.72%	7	CARGMGFGGHYWYFDLW	V6-1 D5-18 J4	-0.094	6.73
	0.72%	8	CTRGWNGVVDEGAFDYW	V1-46 D5-12 J4;V1-3 D5-12 J4	-0.470	4.03
	0.62%	9	CARPLSLVPAAEHNWFDPW	V4-39 D2-2 J5	-0.163	5.32
	0.51%	10	CARDRPNGDQGDFDYW	V3-48 D2-8 J4;V3-53 D2-8 J4	-1.719	4.14
	0.51%	11	CARGGFGSTAGLFDYW	V6-1 D3-10 J4	0.138	5.83
	0.51%	12	CARITHTYDILTGHYIDYW	V2-70D D3-9 J4	-0.189	5.98
	0.51%	13	CASPPMVRGAWMDVW	V5-51 D3-10 J6	0.273	5.83
ZHG	4.49%	1	CAKFGDWDGTDIYYGMDVW	V1-8 D2-21 J6;V1-8 D2-21 J6	-0.342	3.77
	2.57%	2	CVALGIDGLPNW	V1-2 D6-13 J4	0.858	3.8
	2.41%	3	CARGMRDAFDTW	V6-1 D1-1 J3;V4-59 D1-1 J3	-0.6	5.95
	1.93%	4	CAHRRQLGSHFDYW	V2-5 D6-13 J4;V2-70 D6-13 J4	-1.064	8.24
	1.61%	5	CAHRRLGVPAAGAFDPW	V2-5 D6-13 J5;V2-70 D6-13 J5	-0.006	8.26
	1.12%	6	CARDTARGPDDPRFDSW	V1-69 D3-16 J5	-1.447	4.58
	1.12%	7	CARSVDGFDYW	V2-70 D5-12 J4;V2-5 D4-17 J4	-0.327	4.21
	0.96%	8	CAHRHRIPAAGNCFDYW	V2-5 D6-13 J4;V2-70 D6-13 J5	-0.524	8.08
	0.96%	9	CARAVGYGALDW	V3-74 D4-17 J5;V3-23 D4-17 J5	0.408	5.83
	0.96%	10	CARDGRLNWFDPW	V3-48 D5-12 J5;V1-69 D5-12 J5	-0.954	5.95
	0.80%	11	CAHRQSTWDTVDYW	V2-5 D2-15 J4	-1.071	5.21
	0.80%	12	CAREGTSTEAQLGYYYGMDVW	V1-46 D1-26 J6;V1-8 D1-26 J6	-0.510	4.14
	0.80%	13	CARGGPLDNPLQPAAMSDALDVW	V1-69 D2-2 J3	-0.091	3.93
	0.80%	14	CARWYHNNWAFDSW	V1-18 D1-1 J5;V1-69 D1-1 J5	-1.007	6.73
	0.80%	15	CVRDGGRSAFWSAYYTPQDVFDMW	V1-69 D3-3 J3	-0.421	4.43
	0.64%	16	CAKNVVYGTTWYGYHDYW	V3-23 D6-13 J4;V3-48 D6-13 J4	-0.589	6.73
	0.64%	17	CARFGDWDGYDYHYGMDVW	V1-18 D2-21 J6;V1-46 D2-21 J6	-0.811	4.13
	0.64%	18	CARGGPLDNPLQPAAMSDAMDVW	V1-69 D2-2 J3	-0.174	3.93
	0.64%	19	CARLQVVYGGDPADWYFDLW	V5-51 D4-23 J2	-0.02	3.93
	0.64%	20	CARQIYGGVDYW	V6-1 D3-3 J5	-0.233	5.83
LHG	22.61%	1	CARGSPFLDSW	V4-30-2 D4-23 J4;V4-59 D4-23 J4;V4-39 D4-23J4; V4-31 D4-23 J4;V4-34 D2-21 J4;V4-4 D4-23 J4; V4-61 D4-23 J4; V4-30-4 D4-23 J4	-0.145	5.83
	8.49%	2	CATGWLRSYFDLW	V6-1 D5-12 J4/V4-59 D5-12 J4/V6-1 D5-12 J4	0.131	5.83
	4.97%	3	CARGSPFLDYW	V4-30-2 D4-23 J4;V4-61 D4-23 J4;V4-59 D4-23J4; V4-39 D4-23 J4;V4-34 D2-21 J4;V4-30-4 D4-23J4; V4-4 D4-23 J4	-0.191	5.83
	3.85%	4	CATLRQGLASPYYFDSW	V5-51 D6-6 J4/V1-18 D6-6 J4	0.835	5.83
	2.98%	5	CARHAGLRDPKWYSGMGVW	V5-51 D1-7 J6	-0.542	9.31
	2.06%	6	CGSLRLALADPYYFDSW	V5-51 D6-19 J4	0.1	4.21
	1.99%	7	CVSPLGYW	V5-51 ND J4	0.688	5.52
	1.86%	8	CARSIPVAAPTHFDFW	V5-51 D6-19 J4	0.338	6.74
	1.13%	9	CGRHSLPRAVLDSTNWAQFEDW	V5-51 D6-13 J4	-0.686	5.38
	1.06%	10	CARQRYGGYYGSGSYGPYYSMDAW	V4-39 D3-10 J6;V4-31 D3-10 J6;V4-30-2 D3-10 J6; V4-59 D3-10 J6;V4-30-4 D3-10 J6	-0.946	8.12
	1.06%	11	CARQRYGGYYGSGSYGPYYSMDVW	V4-59 D3-10 J6;V4-39 D3-10 J6;V4-30-2 D3-10 J6	-0.846	8.12
	0.99%	12	CARGGPSVDVW	VV4-30-2 D3-10 J6;V4-31 D3-16 J3;V4-4 D3-16 J6	0.055	5.83
	0.92%	13	CSGGGLVRGDIHWFDPW	V6-1 D3-10 J5	-0.159	5.21
	0.72%	14	CARLGREWEPPYFDSW	V5-51 D1-26 J4	-1.008	4.68
	0.66%	15	CAHRGGGSYEDYW	V2-5 D2-21 J4	-1.223	5.32
	0.66%	17	CARQLTKYYDSDIPYQSGLDAFDVW	V5-51 D2-21 J3	-0.715	5.95
	0.60%	16	CARDSWDRVFGPW	V4-31 D2-15 J5;V4-59 D2-15 J5;V4-61 D2-15 J5; V4-30-2 D2-15 J5;V4-34 D2-15 J5	-0.532	4.14
	0.60%	18	CARGGSGEIGYFDYW	V3-53 D5-12 J4;V3-11 D5-12 J4;V3-74 D5-12 J4	-0.387	4.37
	0.53%	19	CARDLSGSYFDSW	V4-61 D1-26 J4;V4-30-2 D1-26 J4	-0.431	4.21

**Table 6:** The mutated position in LHG IgG heavy chain CDR3 sequences (CARGSPFLDSW).

Vname	3'V-REGION	N	D-REGION	5'J-REGION	Jname	Dname	Vmut	Dmut	Jmut
**IGHV4-30-2**	tgtgc$\underline{\text{c}}$agag.^a^		ggt$\underline{\text{c}}$g$\underline{\text{c}}$$\underline{\text{c}}$a$\underline{\text{t}}$tcc	ttgact$\underline{\text{c}}$ctgg	IGHJ4	IGHD4-23	1	4	1
**IGHV4-30-4**	tgtgccagag.		ggt$\underline{\text{c}}$g$\underline{\text{c}}$$\underline{\text{c}}$a$\underline{\text{t}}$tcc	ttgact$\underline{\text{c}}$ctgg	IGHJ4	IGHD4-23	0	4	1
**IGHV4-31**	tgtgc$\underline{\text{c}}$agag.		ggtcgccattcc	ttgact$\underline{\text{c}}$ctgg	IGHJ4	IGHD4-23	1	4	1
**IGHV4-34**	tgtgc$\underline{\text{c}}$agagg	gtc	……gc$\underline{\text{c}}$attcc	ttgact$\underline{\text{c}}$ctgg	IGHJ4	IGHD2-21	1	1	1
**IGHV4-59**	tgtgc$\underline{\text{c}}$agag.		ggt$\underline{\text{c}}$g$\underline{\text{c}}$$\underline{\text{c}}$a$\underline{\text{t}}$tcc	ttgact$\underline{\text{c}}$ctgg	IGHJ4	IGHD4-23	1	4	1
**IGHV4-61**	tgtgccagag.		ggt$\underline{\text{c}}$g$\underline{\text{c}}$$\underline{\text{c}}$a$\underline{\text{t}}$tcc	ttgact$\underline{\text{c}}$ctgg	IGHJ4	IGHD4-23	0	4	1
**IGHV4-61**	tgtgc$\underline{\text{c}}$aga.	g	ggt$\underline{\text{c}}$g$\underline{\text{c}}$$\underline{\text{c}}$a$\underline{\text{t}}$tcc	ttgact$\underline{\text{c}}$ctgg	IGHJ4	IGHD4-23	1	4	1

^a^ Red bases indicate the mutate bases in CDR3 region.

## Discussion

Theoretically, B lymphocyte diversity in each individual is approximately 10^11^ types [18]. However, due to bias in each accession of families, the estimated B lymphocyte BCR in the circulatory system of a healthy individual is found to be approximately 10^6^ types [19,20], which is still a very large number. It is a challenge to completely identify the detailed characteristics of BCR repertoire. In the recent 5 years, HTS has been able to preliminarily assess the basic BCR repertoire characteristics of an individual in a specific time point to: (1) estimate the size and changes in the overall BCR repertoire of an individual, (2) show the uniqueness of BCR repertoire in different B lymphocyte subsets, (3) compare variations in the BCR repertoires between elderly and youth in response to the same vaccine, (4) show characteristics of humoral immune response towards different antigens (e.g., bacterial antigens, viral antigens, and autoimmune antigens) and to search for high affinity antibodies against different antigens, and (5) monitor the lymphoma, minimal residual disease, and immune reconstitution after hematopoietic stem cell transplantation via B lymphocyte cloning to further diagnose and monitor the diseases [21].

B lymphocyte immune response of an individual to vaccine has been the key in the development of immuno-protective effects. Galson et al. conducted a systemic overview of human B lymphocyte receptor library against influenza, tetanus, B hemophilus influenza type, streptococcus pneumonia, rotavirus, human immunodeficiency virus, hepatitis C virus, cytomegalovirus, Epstein-Barr virus, staphylococcus aureus, and dengue virus vaccines [22]. The results showed that BCR repertoires can be used in the vaccine-induced immune responses. Recombinant HBV vaccine has been widely used in the world. Immunization with recombinant HBV vaccine can induce specific immune response to generate B lymphocytes secreting protective HBsAb and form memory B lymphocytes, which becomes the major therapy against HBV in humans. Galson et al. analyzed IgG and IgM heavy chain repertoires from circulating B cells before and after HBV vaccination using Illumina sequencing, their results showed the HTS could help us to understand immune responses to disease and vaccination, they also reported that the repertoires appear to correlate with the PCs appearing at 7 days after immunization, and has a more similar kinetic to appearance of memory cells at 21 days after immunization [12]. They also reported that focusing on the vaccine-specific repertoires and subsequent doses were essential to find high affinity B cell clones to specific antigen [13]. But there is no research to analyzed the compositions and characteristics of IgM and IgG chain CDR3 repertoires. However, the heterogeneity of B lymphocytes secreting HBsAb in the human body, and their characteristics as well as the life-span of memory B lymphocytes have not been fully elucidated.

In the present study, we used Roche 454 GS-FLX-HTS to reveal BCR H chain CDR3 repertories in 3 healthy volunteers before and after immunization with recombinant HBV vaccine, and comparatively analyze the formation and characteristics of IgM and IgG H chain CDR3 repertoires. Our findings showed that IgG H chain CDR3 repertoires diversity was significantly reduced compared to IgM H chain CDR3 repertoires in the healthy volunteers after immunization with recombinant HBV vaccine, suggesting that immunization with recombinant HBV vaccine might induce individual’s B lymphocytes to generate IgG-based immune response. Truck et al. proposed the use of HTS to detect high-frequency common sequences among different samples to screen antigen-specific B lymphocytes. In this study, the HBsAb levels in the healthy volunteers six-month after immunization with recombinant HBV vaccine were higher than the HBsAb levels in normal immune response (> 100 IU/L). However, none of the individuals showed single or unique high-frequency CDR3 sequences after immunization, but multiple high-frequency CDR3 sequences. This suggests that the current recombinant HBV vaccine contains multiple HBsAg conformational epitopes that can trigger humoral immune response, and it can activate multiple B lymphocyte clones in the body. These findings were consistent with a previous study on sequence analysis of the monoclonal antibody, which specifically bound to HBsAg. Tajiri et al. analyzed 31 sequences of HBsAb, where 5 source sequences were of memory B cells, and 26 source sequences of plasma cells. These sequences had high affinity for HBsAg. Furthermore, the family source sequences and CDR3 sequences demonstrated heterogeneity [23]. Our findings also consistent with Chang’s report, they used HTS to characterize the H chain CDR3 sequences in paired siblings of 4 families in which only one member of each pair had chronic HBV infections, they reported that vaccination induced significant increases of H chain CDR3 cluster diversities among siblings without hepatitis B [24]. Our studies showed that the 3 healthy individuals shared a small number of CDR3 sequences before and after the immunization. However, we did not find identical IgG or IgM H chain CDR3 amino acid sequence among the 3 healthy volunteers. This might be due to differences in the HLA-DR site, antigen presentation, and helper T cell-activated B lymphocytes in the 3 individuals. Our findings suggested that although the 3 individuals generated protective HBsAb with high titers, the HBsAb had great heterogeneity in different individuals and originated from multiple B lymphocytes clones. Truck et al. have previously proposed public sequences as the sequences those are constituted by the same CDR3 amino acids in at least 2 individuals [14]. The current findings suggested that immunization with the same epitope of antigen might not yield the same CDR3 amino acid sequence in the body of multiple individuals.

Several studies have analyzed the high-frequency CDR3 mechanism and significance after immunization with the vaccine in humans. Jiang et al. studied human IgG H chain CRD3 after immunization with the flu vaccine, and demonstrated a high-frequency amplification of high-affinity sequence against the flu vaccine [25]. In this study, we screened the IgG H chain CDR3 high-frequency amino acid sequence (> 0.5%) in the 3 healthy volunteers after immunization with recombinant HBV vaccine, and analyzed the compositional characteristics of CDR3 high-frequency amino acid sequence. We found that the same CDR3 amino acid sequence can be formed by the rearrangement of multiple V-D-J families. This phenomenon was common in the all the healthy volunteers. Five out of 13 sequences in volunteer Y, 12 out of 20 sequences in volunteer L, and 10 out of 19 sequences in volunteer Z were multiple V-D-J family sources. The same antigenic epitope probably selected different B lymphocyte clones for clonal expansion, and through somatic hypermutation produced identical CDR3 amino acid sequence with high affinity. We studied the IgG H chain CDR3 high-frequency amino acid sequence in volunteer L (LHG) as an example (CARGSPFLDSW), using an integrated software IMGT/V-quest to analyze the CDR3 regional mutations of LHG CARGSPFLDSW (red text in Table 6 represents the bases in the mutation). The V gene family of LHG CARGSPFLDSW originated from IGHV4-30-2, IGHV4-30-4, IGHV4-31, IGHV4-34, IGHV4-59 and IGHV4-61; the D gene family originated from IGHD4-23 and IGHD2-21; and the J gene family originated from IGHJ4. Some families showed single point mutation and other families demonstrated multiple point mutations. We analyzed the high-frequency CDR3 of multiple V-D-J origins to obtain similar mutation results. Relationship between high-frequency amino acid sequence of IgG H chain CDR3 and HBsAg affinity after immunization with HBV vaccine needs further verification. In addition, we obtained large differences in individual B lymphocyte response levels and memory B lymphocyte maintenance after immunization with recombinant HBV vaccine. The lack of T/B lymphocytes in some families resulted in 5–10% individuals unresponsive to the recombinant HBV vaccine [26]. Most individuals demonstrated a gradual decline in the levels of HBsAb, with eventually no immuno-protective effects in approximately 10 years after immunization with recombinant HBV vaccine. However, none of the studies clearly elucidate the number and the maintenance of HBsAg-specific memory B cells. The results from this study suggested that detection of IgG H chain CDR3 repertoires compositional characteristics, especially the changes of high-frequency CDR3 amino acid sequence when monitoring the HBsAb level after immunization with recombinant HBV vaccine provide basic data and new research strategies to study B lymphocyte heterogeneity and memory B lymphocyte maintenance in humans.

## Supporting Information

S1 File Raw sequences.zip. The raw sequences obtain from Tongji-SCBIT Biotechnology Corporation. The 6 samples from Y’s pre-immunization, Y’s post-immunization, L’s pre-immunization, L’s post-immunization, Z’s pre-immunization, and Z’s post-immunization were labelled as MID1 = YQ, MID2 = YH, MID3 = LQ, MID4 = LH, MID5 = ZQ, and MID6 = ZH, respectively.
(ZIP)
